# Mitigation of cadmium toxicity in African catfish using biological Nano chitosan: insights into biochemical, genotoxic, and histopathological effects

**DOI:** 10.1186/s12917-025-04673-4

**Published:** 2025-04-16

**Authors:** Dalia H. Samak, Hoda A. Abd-Ellatieff, Riad H. Khalil, Nehad A. Saleh, Hamida M. Saleh

**Affiliations:** 1 Department of Forensic Medicine and Toxicology, Faculty of Veterinary Medicine, Damanhour University, El-Beheira, Egypt; 2 Department of Pathology, Faculty of Veterinary Medicine, Damanhour University, El-Beheira, Egypt; 3 Department of Poultry and Fish Diseases, Faculty of Veterinary Medicine, Alexandria University, Alexandria, Egypt; 4 Department of Animal Hygiene, Faculty of Veterinary Medicine, Damanhour University, El-Beheira, Egypt

**Keywords:** Cadmium, Toxicology, Catfish, Bio-CHNPs, Biochemical, Antioxidant defense, Inflammation, Pathology

## Abstract

**Background:**

Cadmium is a highly toxicant heavy metal that poses serious risks to aquatic organisms, animals, and humans. Recent studies have investigated using biological chitosan nanoparticles (Bio-CHNPs) as a potential solution to alleviate the harmful effects of Cd exposure, particularly in aquaculture. Bio-CHNPs have gained attention for their applications in drug delivery and biomedical research, indicating their potential utility in addressing environmental toxicity.

**Objective:**

This research aims to explore the effectiveness of Bio-CHNPs in mitigating cadmium chloride (CdCL_2_) toxicity in African catfish (*Clarias gariepinus).*

**Methods:**

One hundred and twenty (n = 120) catfish were divided into 4 groups; G1 (control); G2, intoxicated with 10% LC_50_ of CdCL_2_; G3 received 3 g/kg of Bio-CNPs; G4, treated with 10% LC_50_ of CdCL_2_ and Bio-CNPs 3 g/kg feed.

**Results:**

CdCl_2_ exposure resulted in severe liver, intestine, and kidney damage, which was evidenced by alterations in biochemical parameters, hormonal imbalance, DNA damage, and micronucleus formation. Antioxidant defense mechanisms were compromised, as the activities of Superoxide Dismutase (SOD), Total Antioxidant Capacity (TAC), and Catalase (CAT) were reduced. mRNA expression levels of inflammatory cytokines such as IL-1β, IL-8, and LBP were also significantly elevated following CdCl_2_ exposure. Conversely, Bio-CHNPs treatment showed antioxidant and anti-inflammatory effects, greatly lowering the biochemical, genotoxic, and histopathological effects induced by CdCl_2_.

**Conclusion:**

The outcomes of this study are indicative of the potential of Bio-CHNPs as a promising aquaculture feed supplement, with a dual advantage of antagonizing the toxicity of environmental pollutants like Cd and imparting antioxidant and immunomodulatory effects. Bio-CHNP supplementation can be a viable strategy for remedying aquatic environmental heavy metal pollution, with the ultimate safeguarding of human health and ecosystem balance.

**Supplementary Information:**

The online version contains supplementary material available at 10.1186/s12917-025-04673-4.

## Introduction

The growth of aquaculture in Egypt is affected by extremely serious challenges mainly on the basis of water pollution of natural water bodies, including rivers, lakes, and canals. The pollution of water is mainly due to chemical products manufactured by human beings, which exert significant impacts on water quality for aquaculture and disrupt aquatic ecosystems [1].

Catfish have a long history of propagation and management that dates back to the early twentieth century [2]. They pose a crucial role in freshwater aquaculture, accounting for over half of the industry [3]. Moreover, channel catfish are highly valued as sportfish and are commonly found in waterways throughout North America [4] Their unintentional introduction outside their native habitats has prompted global research interest [5]. As a result, channel catfish represent an ideal model species for assessing the impacts of cadmium exposure, thanks to their extensive distribution and well-documented physiology [6, 7] and the potential economic and ecological implications of insights gained from such studies.

Fish are highly susceptible to Cadmium (Cd) contamination in the environment due to multiple exposure routes, including direct absorption from water through divalent metal transporters and ingestion through their diet [8].

Cadmium is a very toxic metal with a high risk to aquatic ecosystems and human health [9]. It accumulates in aquatic organisms and persists in the food chain as it is non-destructed. It has a large biological half-life of 16–30 years in humans, resulting in prolonged toxic effects [10–14]. Cd exposure severely compromises tissue structure and function, disrupts the antioxidant defense system, and negatively affects reproductive regulation and immune responses in fish depending on Cd concentration and duration of exposure [15]. Furthermore, consumption of Cd-contaminated fish can lead to various health issues in humans as Cd is toxic to the immune and nervous systems, genes, kidneys, and cells, and has the potential to induce cancer in various organisms, including humans [13, 16, 17].

Chitosan nanoparticles (CH-NPs) are positively charged polymeric nanoparticles derived from chitin in the shells of crustaceans, which have emerged as a multifunctional and versatile material with immense applications [18]. CH-NPs are biodegradable, biocompatible, non-toxic, and environmentally friendly and thus find applications in wide-ranging fields such as agriculture, medicine, and pharmacy [18]. There are some methods used for CH-NPs production like emulsification & crosslinking, ionic gelation, and precipitation-based approaches, however, the use of physical and chemical approaches has many disadvantages which are the use of high-pressure, temperature, energy, harmful chemicals, and the large particles size [18]. Therefore, the use of green approaches in the synthesis of CH-NPs was utilized to produce ultrafine nanoparticles with other advantages such as a highly reduced size of NPs of less than 100 nm, which is a significant characteristic for most applications where the specific surface area is involved. Microorganisms such as fungi and bacteria are utilized for nanoparticle biosynthesis [19].

Due to The unique biological activities and properties of CH-NPs as anti-inflammatory, antioxidant, and ROS scavenging agents make them extremely useful for pharmaceutical and biomedical applications and also for the mitigation of heavy metal toxicity in aquatic animals such as catfish [20]. Therefore, in the current study, it was proposed to examine the effect of the addition of CH-NPs synthesized by *Bacillus subtilius* (Bio-CHNPs) on the biochemical, physiological, immune response, oxidative stress, genotoxicity and pathological changes induced by CdCL_2_ toxicity in catfish.

## Materials and methods

### Ethical approval

The study adhered to ethical roles approved by the Institutional Animal Care and Use Committee (IACUC) of Damanhhour University, specifically from the Faculty of Veterinary Medicine, Department of Forensic Medicine and Toxicology. (approval number**: (DMU/VetMed-2024/019).** The trial lasted for 8 wks and adhered to international ethical standards. No human participants were involved, and all experimental groups were provided with a basal diet.

### Preparation of biological chitosan nanoparticles by *Bacillus subtilius*

Bio-CHNPs were synthesized using *Bacillus subtilis* and chitosan. The process involved incubating *Bacillus subtilis* with chitosan in Enrichment Medium broth, leading to CHNP biosynthesis indicated by a color change. Bio-CHNPs were then synthesized from *Bacillus subtilis* pellets through autoclaving and centrifugation. The conditions for Bio-CHNPs biosynthesis included Trypticase Soya broth, temperatures of 25 °C and 30 °C, reaction times of 1 and 2 days, and agitation speeds of 200 and 220 rpm, with a pH range of 8 and 9 [21].

### Characterization of biological chitosan nanoparticles

Transmission electron microscopy (TEM) images and Zeta sizer analyzer and Zeta potential obtained at the Central Laboratories, City of Scientific Research and Technological Applications, Alexandria, Egypt, were utilized to detect the size and average shape of Bio-CHNPs.

### Antioxidant activity (DPPH assay) of biological chitosan nanoparticles

The Bio-CHNPs radical scavenging activity was detected as described earlier with few modifications [22]. Absorbance was read at 517 nm after 30 min using a microtiter plate reader (BioTek Elx808, USA) using Eq. (1) as Radical scavenging activity (%) = (control absorbance—sample absorbance) / (control absorbance) × 100.

### ABTS assay of biological chitosan nanoparticles

The Bio-CHNPs antioxidant activity was done as outlined by Gil et al. [23]. Absorbance was estimated at 745 nm after 30 min, using a microtiter plate reader. TBHQ and ABTS were used as controls. % RSA Inhibition = (A _control_—A _sample_) / (A _control_) × 100.

### Antibacterial activity of biological chitosan nanoparticles

The antibacterial activity of Bio-CHNPs was recorded through the disc diffusion method [24]. Approximately 100 μl of bacterial inoculum (1 × 10^8 CFU/ml) was spread on the surface of MHA plates. Sterilized paper discs (5 mm in diameter) were soaked with various concentrations of Bio-CNPs (1, 2, and 3 μg/ml) and placed around the edges of the MHA plates. Control discs were treated with sterilized distilled water. The plates were incubated at 37 °C for 24 h. The inhibition zones around the discs were then measured in millimeters.

### Experimental setup and fish rearing

The toxicant substance, CdCl_2_; purity > 95% and molecular weight: 183.32 was obtained from Al-Gomohria Co. in Alexandria, Egypt. A fresh stock solution of Cd was prepared by dissolving CdCl_2_ in deionized water to achieve a concentration of 1000 mg Cd /L. A total of 120 African catfish (*Clarias gariepinus*), each with an average weight of 150 ± 20 g, were purchased from a private Fish Farm located in Kafr-Elsheikh Governorate, Egypt. The healthy fish were quickly transported via plastic bags to the Forensic Medicine and Toxicology laboratory at the Faculty of Veterinary Medicine, Damanhour University/ Egypt, and acclimated for two weeks in glass aquaria (40 × 35 × 70 cm) with a capacity of 60 L each. During this acclimation period, the fish were fed a standard fish diet [25]. Aquaria were provided with air pumps for improving aeration. Fish were given a basal control diet with 25% crude protein (CP) three times a day until satiety was apparent. A twelve-hour cycle of dark and light was established using a fluorescent light cylinder. The dissolved oxygen levels, pH, and water temperature were checked daily using a particular portable equipment [26]. In addition, the levels of unionized ammonia were estimated twice a day using an automated probe [27]. Fish debris and waste were siphoned out every two days, and half of the glass aquaria water was replaced with fresh dechlorinated water having the same Cd conc (semi-static method). Daily monitoring of water quality parameters was done [25]. In the semi-static approach, 50% of the water was replaced, and the required Cd concentration was supplemented. Cd concentrations were verified using gas chromatography-mass spectrometry (GC–MS). This ensures homogeneity in each glass aquarium. At the end of the acclimatization period, the catfish was randomly divided into four equal groups, with 10 fish in each aquarium. Each group consisted of triplets, resulting in 30 fish for each treatment. Groups were divided as follows; G1 (NC, Control negative group); G2, intoxicated with 10% LC_50_ of CdCL_2_ (5.64 mg/l) [28]; G3 received 3 g/kg of Bio-CNPs; G4, treated with 10% LC_50_ of CdCL_2_ and supplemented with Bio-CHNPs for 8 weeks wks.

### Sampling

At the 8th Wks following CdCl_2_ exposure and Bio-CHNPs supplementation, fish were sampled. Before sampling, the fish were fasted for 24 h and then anesthetized by placing them in aquaria with clove oil at 50 µl/L for five minutes. Blood samples were taken from the caudal vein using a 3 ml syringe, ensuring the procedure took less than 3 min to reduce handling stress. The blood samples were then split into two tubes: one with heparin as an anticoagulant and one without for biochemical analysis and genotoxicity, respectively. Samples were preserved at 4 °C. Liver tissues were collected for gene expression analysis, with some portions stored at −80 °C and others preserved in formalin 10% for histopathological examination.

### Hepatic, and renal cadmium residue analysis

Following decapitation, Hepato-renal tissues were taken from the fish and converted to freeze-drying. The tissues underwent a thorough washing process in fresh water, followed by a brief rinse in double-deionized water to remove residual contaminants. Excess water on the liver and kidney surface was dried by blotting with filter paper. Subsequently, the tissues were dried overnight at 80 °C and weighed (approximately 0.3 g dry weight). After weighing, the tissues were digested and filtered according to the method outlined by Allen et al., [29]. The filtrate was diluted with double-deionized water and measured using atomic absorption.

### Biochemical parameters analysis

Serum biochemical assays which include aspartate aminotransferase (AST) and alanine aminotransferase (ALT) were determined calorimetrically according to Reitman et al. [30] using (Biodiagnostic Co., Cairo, Egypt) kits according to the manufacturer's guidelines. Glucose levels were determined using glucose enzymatic PAP kits [31]**.**

### Immune assay

Respiratory burst (RB): system failure. RB was evaluated using bitrotetrazolium blue chloride (NBT) [32]. Serum lysozyme activity was evaluated and measured spectrophotometrically [33]. The total serum immunoglobulin (Ig) and Protein levels were estimated using ELISA kits [34].

### Physiological and hormone assay

Stress-related hormones, including cortisol, were assessed using the methods of Sadoul and Geffroy [35]. The growth-related hormones, such as growth hormone and glucose levels were also measured following the PTrinder method [36]. Reproductive-related hormones, including follicle-stimulating hormone, testosterone, and progesterone, were measured using ELISA following the manufacturer’s kit instructions [37].

### Antioxidant status evaluation

Liver and kidney samples were cleaned and prepared (6 fish/treatment). The sample tissues (10 mg/each organ) were homogenized in buffered phosphate saline (PBS, pH 7.8) and then centrifuged at 10.000 rpm for 30 min. Superoxide dismutase (SOD) was measured following Islam et al., [38]. Catalase (CAT) was determined following Tehrani et al., [39]. Total antioxidant capacity (TAC) according to the protocol given by Gupta et al., [40]. Total peroxides (TPX) follow Sayed et al., [41].

#### mRNA expression of genes related to antioxidant and inflammatory pathways

At the end of the experiment, hepatic tissues were collected and processed for mRNA extraction using the PureLink™ RNA Mini Kit (Invitrogen, Fair Lawn, NJ, USA) according to the manufacturer’s protocol. The extracted RNA was quantified with a NanoDrop™ 2000 spectrophotometer (Thermo Scientific, Wilmington, NC, USA). CdNA was then synthesized using the iScript™ CdNA Synthesis Kit (BIO-RAD, Hercules, CA, USA), following the manufacturer's instructions. For qPCR analysis, reactions were carried out using the CFX Connect™ Real-Time PCR System (BIO-RAD) with iTaq Universal SYBR Green supermix 2X (BIO-RAD) and specific primers for genes such as Interleukin 1β (IL1β), Interleukin 8 (IL8), lipopolysaccharide-binding protein (LBP), Glutathione S-transferase (GSTa), Glutathione peroxidase (GPX), and Glutathione-disulfide reductase (GSR) (Supplementary Table 1). The qPCR was performed in triplicate with 100 ng of CdNA and 400 nM of primers. Gene expression levels were quantified using the 2 − ΔΔCt method and a standard curve and the data of relative mRNA expression were analyzed [42].

### Determination of DNA damage at the single-cell level

All procedures were done under dim light conditions and at 4 °C to prevent DNA photo-oxidation. Two slides per fish were prepared for each fish, with 100 "comets” cells examined per slide. Images of the non-overlapping comet cells were captured with a camera and analyzed using CASP computer software [43]. The tail DNA percentage (% tail DNA) was recorded as an indicator of DNA damage. DNA fragmentation was calculated as the following DNA fragmentation = Absorbance of fragmented DNA (represented by supernatant) / [Absorbance of fragmented DNA (represented by supernatant) + Absorbance of intact DNA (represented by pellet)] × 100. [44].

### Micronucleus test: assessment of genotoxicity

After collecting blood samples, smears were prepared on clean frosted glass slides and dried in the air at room temperature. The slides were then fixed for 10 min and stained with 6% Giemsa solution in buffer (pH 6.8) for 30 min. Micronuclei (MNi) scoring was performed using a light microscope (Leitz Wetzlar, Germany, 100X with oil). A total of 10,000 erythrocytes were examined per concentration, and micronucleus bodies were identified [45]. A Micronucleus frequency was evaluated from the formula: MN% = Number of cells containing micronucleus/total of number cells counted × 100.

### Histopathological alterations

Hepatic, Renal, and intestinal tissues of the control and treated fish were removed, fixed in 10% neutral buffered formalin, dehydrated, embedded in paraffin wax, and processed for routine histological evaluation. Sections (5 μm) were prepared and stained with Hematoxylin and Eosin (H&E). Each kidney, intestine, and liver sections were examined in six randomly selected fields under × 400 using a Leica DM500 light microscope by experienced pathologists at the Pathology Department/ Faculty of Veterinary Medicine, Damanhour University, Egypt. Histopathological alterations in liver and kidney tissues were also scored [46]**.**

### Statisticgal analysis

Before conducting a one-way analysis of variance (ANOVA) analysis, normality, and homoscedasticity were assessed using IBM SPSS Statistics V22 software. After these preliminary analyses, Duncan's multiple-range test was used to identify differences among treatments, with significance set at P < 0.05. Data were shown as mean ± standard error (SE).

## Results

### Characterization of biological chitosan nanoparticles

**Fig. 1:**
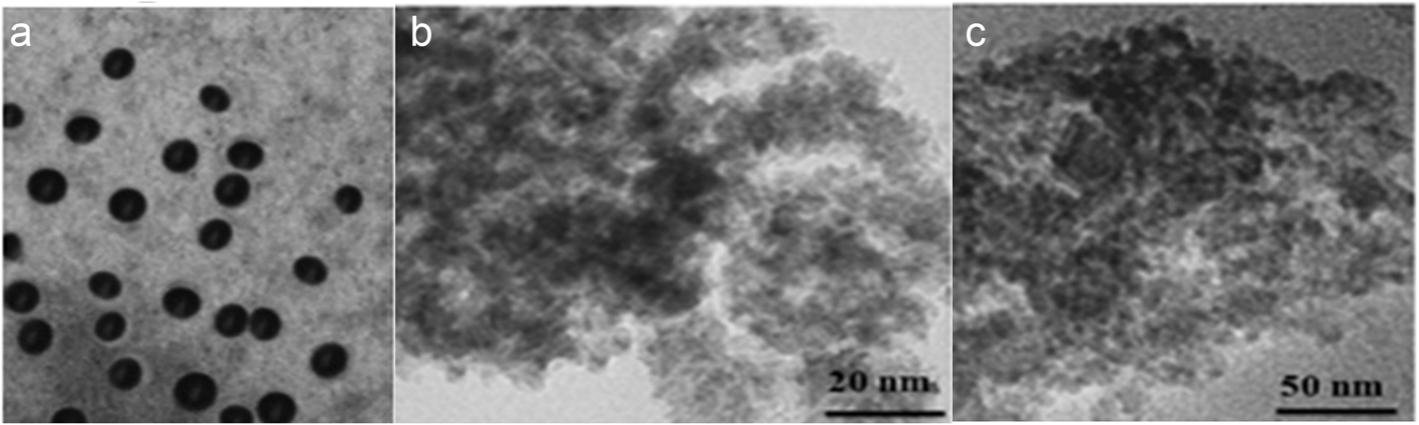
**a** Characterization of biological nano-chitosan (Bio-CHNPs) synthesized *by Bacillus subtilis* the size of 75–81 nm and its shape is spherical in a transmission electron microscopic (TEM) image and concentration 67 μg/ml. (D *Bacillus subtilis* pellets to the chitosan supplemented UV–vis spectrum of chitosan nanoparticles and Particle size and distribution curve for chitosan nanoparticles. (**b**) size and characterization of chitosan nanoparticles biosynthesized agitated at 200 rpm. (**c**) size and characterization of chitosan nanoparticles biosynthesized agitated at 220 rpm using DLC is 8.3 nm.)

Bio-CNPs were relatively spherical, homogeneous by TEM, with a consistent distribution. The particle size ranges between 74 and 84 nm as in (Fig. 1).

### Antioxidant activity of biological chitosan nanoparticles (DPPH assay*)*

Bio-CHNPs exhibited robust radical scavenging activity. This activity potentially reflects the effectiveness of Biological Nano chitosan in neutralizing the free radicals is the best regarding the ascorbic acid and catechin (the commonly known antioxidants), (Table 1).

**Table 1:** Antioxidant activity of biosynthesized chitosan nanoparticles (Bio-CHNPs) synthesized

Items	Radical scavenging activity (%)	% RSA Inhibition
Bio-CHNPs	50.44 ± 2.55a	1.65 ± 0.05a
Ascorbic acid	38.25 ± 4.64b	0.88 ± 0.02b
Catechin	25.42 ± 139c	0.55 ± 0.099c

Values (means±SE) that do not share similar superscripts in the same row differ substantially (One way ANOVA; *P*<0.05)

### Antibacterial activity

Bio-CHNPs exhibit potent antibacterial activity as S*taphylococcus aureus* and *Aeromonas hydrophilia*. The MIC, IZDs, and MBC were greatly increased with the higher concentrations of Bio-CHNPs (1,2 and 3), (Supplementary Fig. 1).

### Clinical signs and survival rate

No deaths or abnormal clinical signs were observed in the negative control group or the Bio-CHNP-supplemented group throughout the experiment. In contrast, fish exposed to Cd exhibited moderate to severe behavioral disorders, including reduced swimming activity, decreased feeding, abnormal swimming behavior (e.g., swimming on their backs), and darkened skin. Notably, Bio-CHNPs supplementation significantly alleviated these behavioral disorders and improved the survival rate of catfish exposed to Cd compared to those only exposed to Cd.

### Cadmium residue analysis in liver, and kidney

**Fig. 2:**
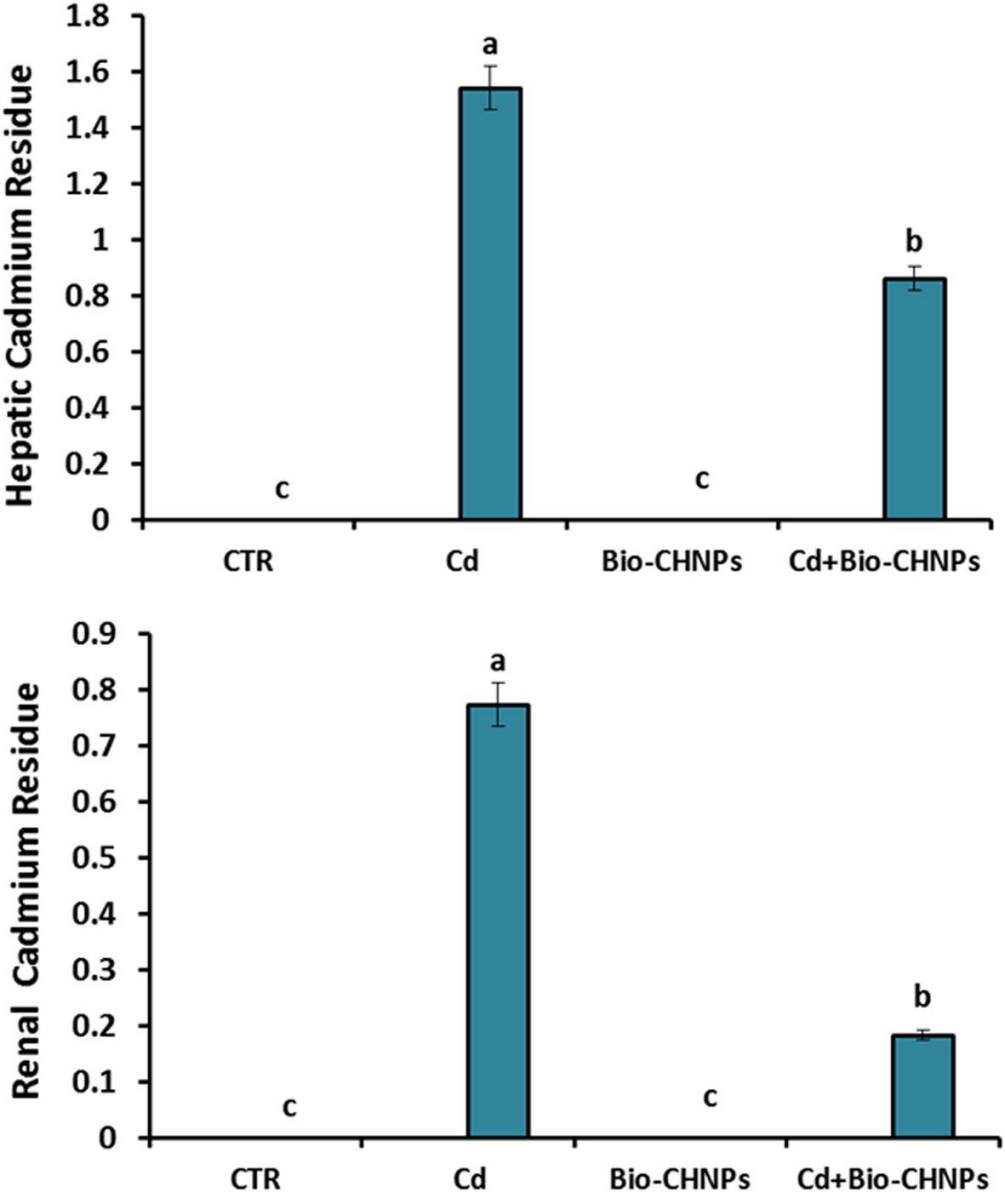
Impact of Bio-CHNPs on cadmium Residue in hepatic and renal tissue of catfish subjected to CdCL2 toxicity for 8 Wks

The concentration of cadmium residue in catfish tissue was significantly influenced by the feeding trial (P < 0.001). In the group fed with Bio-CHNPs alone, no detection of Cd residue was observed. Conversely, the Cd content in hepato-renal fish tissue was statistically elevated in those intoxicated with Cd-polluted water in comparison to all treated groups. However, the biologically Nano chitosan group combined with Cd-polluted water exposure reduced statically in cadmium residue level compared to the group fed a regular diet and reared in Cd-polluted water (Fig. 2).

### Assay of biochemical, immunomodulatory and hormonal parameters

**Fig. 3:**
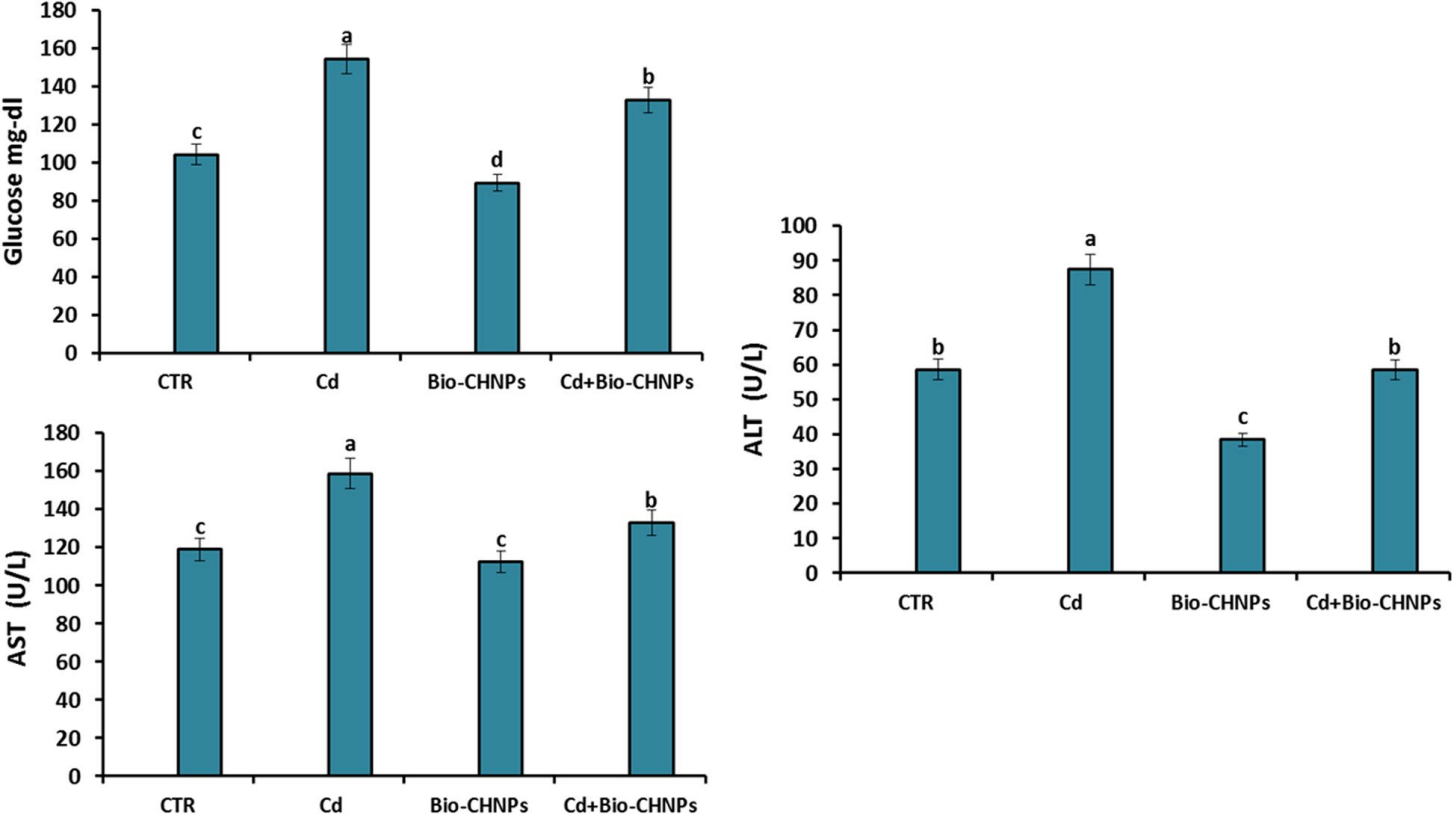
Impact of Bio-CHNPs on hepatic biochemical parameters of catfish subjected to CdCL2 toxicity for 8 Wks. Glucose, ALT; Alanine aminotransferase. AST: aspartate aminotransferase;

Cd- intoxicated fish displayed a noticeable increase (P < 0.001) in hepatic enzyme ALT and AST, with an increase in the glucose levels. Fish supplemented with Bio-CHNPs only (G3) in the absence of heavy metal exposure exhibited normal levels near to the NC (G1). However, Bio-CHNPs treated fish declined the ALT and AST levels compared to the NC (Fig. 3).

**Fig. 4:**
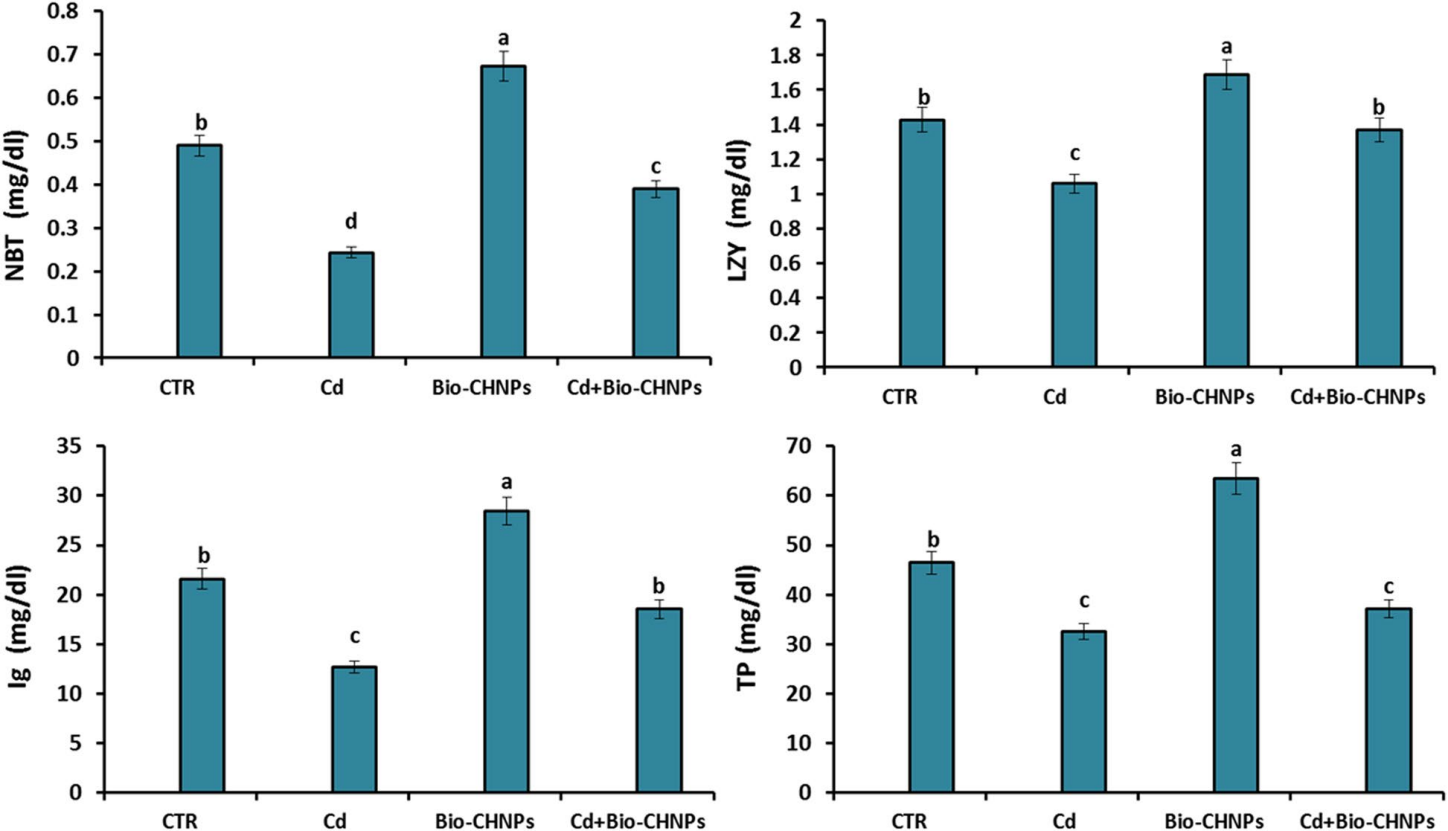
Impact of Bio-CHNPs on immunomodulatory parameters of catfish subjected to CdCL2 toxicity for 8 Wks. Respiratory burst (RB) evaluated using (NBT) bitrotetrazolium blue chloride, LYZ; lysozyme, Ig; immunoglobulin and TP; total protein, Cortisol

Regarding innate immune responses, significant reductions in RB, Ig, TP, and LYZ, were seen in fish exposed to heavy metals as in the G2 group. Supplementation with Bio-CHNPs in Cd-exposed fish (G4) successfully improved the levels of those enzymes and mitigated the adverse effects resulting from exposure to Cd (Fig. 4).

**Fig. 5:**
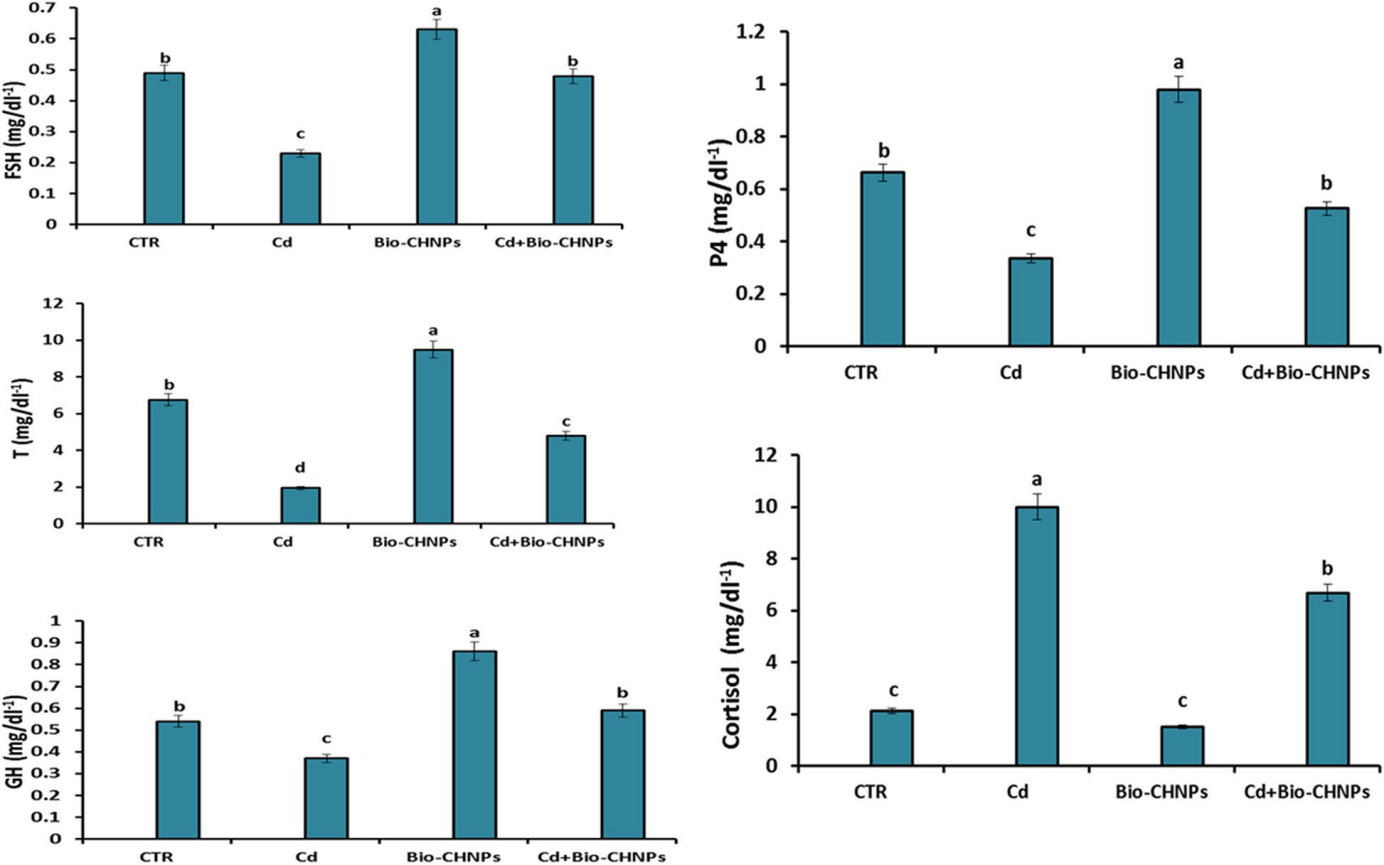
Impact of Bio-CHNPs on hormonal levels of catfish subjected to CdCL2 toxicity for 8 Wks. GH; growth hormone, FSH; follicle-stimulating hormone, T; Testosterone, P4; progesterone

Exposure to Cd-contaminated water significantly reduced levels of follicular stimulating hormone, testosterone, progesterone, and growth hormone in groups G2, while cortisol levels increased in these groups. However, treatment with Bio-CHNPs showed greater improvement and significantly enhanced growth hormone levels and male and female hormone levels, while reducing cortisol levels in groups G4 in comparison with the NC group (G1) (Fig. 5). These findings suggest that the application of Bio-CHNPs in contaminated environments probably enhanced the good effect of biological Nano chitosan against cadmium heavy metals on biochemical parameters in Claris fish.

### Antioxidant markers assay

Cd intoxicated fish in G2 displayed an obvious state of oxidative damage remarked by increased levels of oxidative stress marker as TPX with reduction of antioxidant parameters (SOD, CAT, and TAC), On the contrary, fish treated with Bio-CHNPs in their feed (G4) showed a substantial elevate the level of TAC, CAT, and, SOD with a discernible decrease in TPX as compared to the NC. Bio-CHNPs supplementation in this study may provide effective protection against oxidative stress in fish as depicted in Table 2.

**Table 2:** Effect of heavy cadmium (Cd) exposure and treated with biosynthesized chitosan nanoparticles (Bio-CHNPs) during the 8-weeks experimental period on antioxidants enzymes in liver tissues of Catfish; superoxide dismutase (SOD), catalase (CAT), total antioxidant capacity (TAC), and total peroxides (TPX)

Groups	SOD (IU/L)	CAT (IU/L)	TAC (μM/L)	TPX (μM/L)
CTR	9.67 ± 0.28b	10.5 ± 0.29b	1.27 ± 0.05b	1.48 ± 0.05c
Cd	3.76 ± 0.17d	2.72 ± 0.09d	0.66 ± 0.03c	3.57 ± 0.1a
Bio-CHNPs	13.3 ± 0.17a	14.07 ± 0.11a	1.54 ± 0.06a	1.25 ± 0.04c
Cd + Bio-CHNPs	5.85 ± 0.14c	6.93 ± 0.12c	0.84 ± 0.04c	2.17 ± 0.1b

Values (means±SE) that do not share similar superscripts in the same row differ substantially (One way ANOVA; *P*<0.05)

### Quantitative Real-Time Polymerase Chain Reaction (qRT-PCR) Analysis of antioxidant and inflammatory genes in liver

**Fig. 6:**
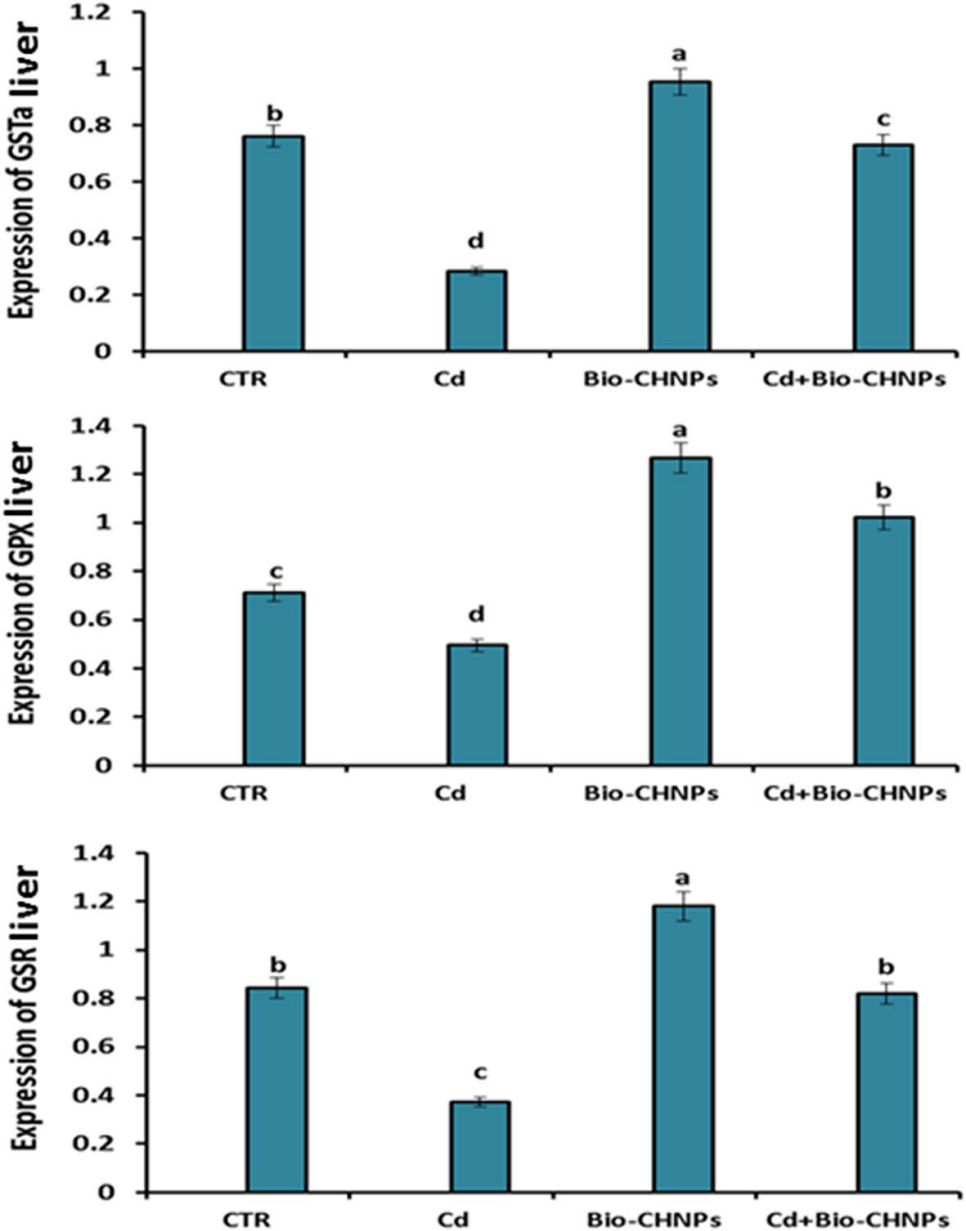
Relative mRNA levels of antioxidant genes in liver tissues of catfish subjected to CdCL2 toxicity for 8 Wks. GST: glutathione S-transferase, GPX: glutathione peroxidase, GSR: glutathione-disulfide reductase

**Fig. 7:**
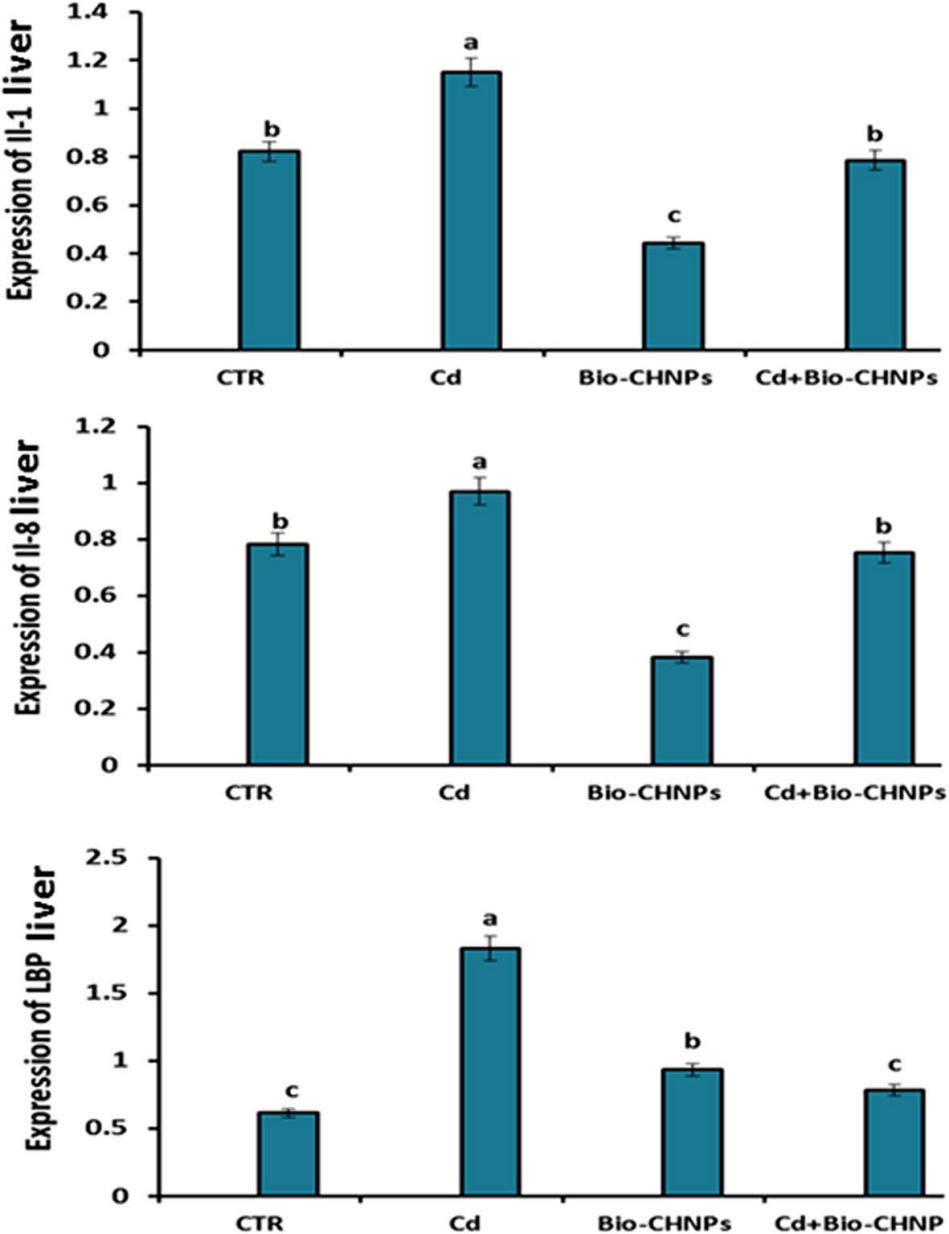
Relative mRNA levels of inflammatory genes in liver tissues of catfish subjected to CdCL2 toxicity for 8 Wks. IL1β: interleukin1β, IL8: interleukin 8, LBP: lipopolysaccharide-binding protein

mRNA Expression of IL8, IL1β, LBP, GST, GSR, and GPx in hepatic tissues of Cd-water intoxication to fish and the group supplemented with Bio-CHNPs upon Cd intoxication are displayed in Fig. 6 and Fig. 7. There was an upregulation in IL1β, IL8, and LBP genes in the liver and kidney of catfish after exposure to Cd intoxication, as compared to the NC group. The higher expression of these genes was suppressed after the Bio-CHNPs administration to Cd-exposed fish (G2). Likewise, the downregulation of GST, GSR, and GPX levels was noted in Cd-intoxicated fish (G2) relative to the NC group. Remarkably, treatment with Bio-CHNPs showed noteworthy betterment in the increased expression of these antioxidant genes either in Bio-CHNPs (G3) or Bio-CHNPs supplemented group upon Cd exposure (G4).

### DNA damage and genotoxicity analysis

**Fig. 8:**
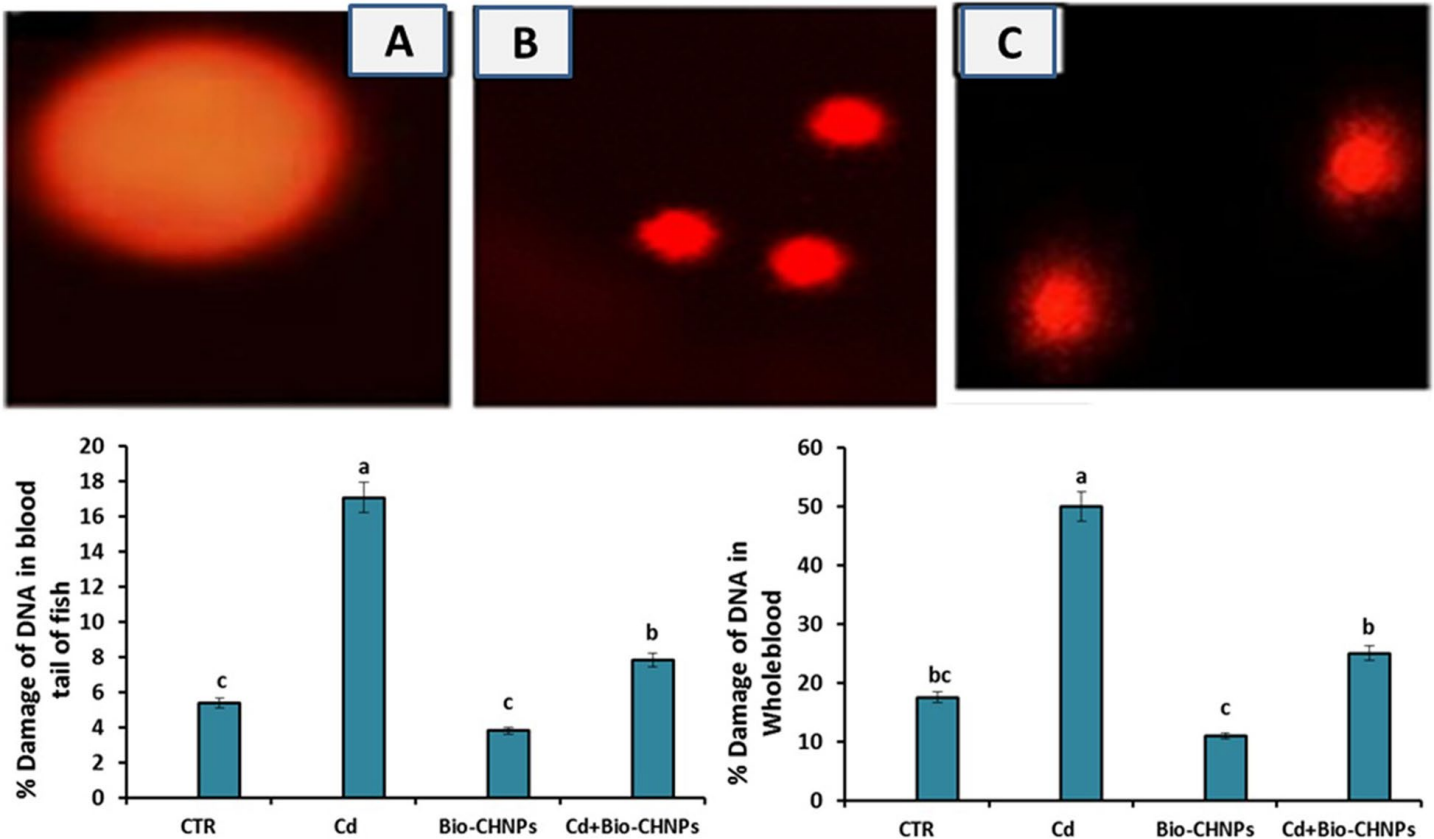
**A, B **and** C **Blood cells of catfish with DNA damage (% Tail DNA) were observed by the comet assay (Magnification 200 ×); pictures are illustrative; the time of exposure is 8 weeks. Electropherogram of blood proteins of the catfish after Cadmium chloride exposure and treated with Bio-CHNPs

**Fig. 9:**
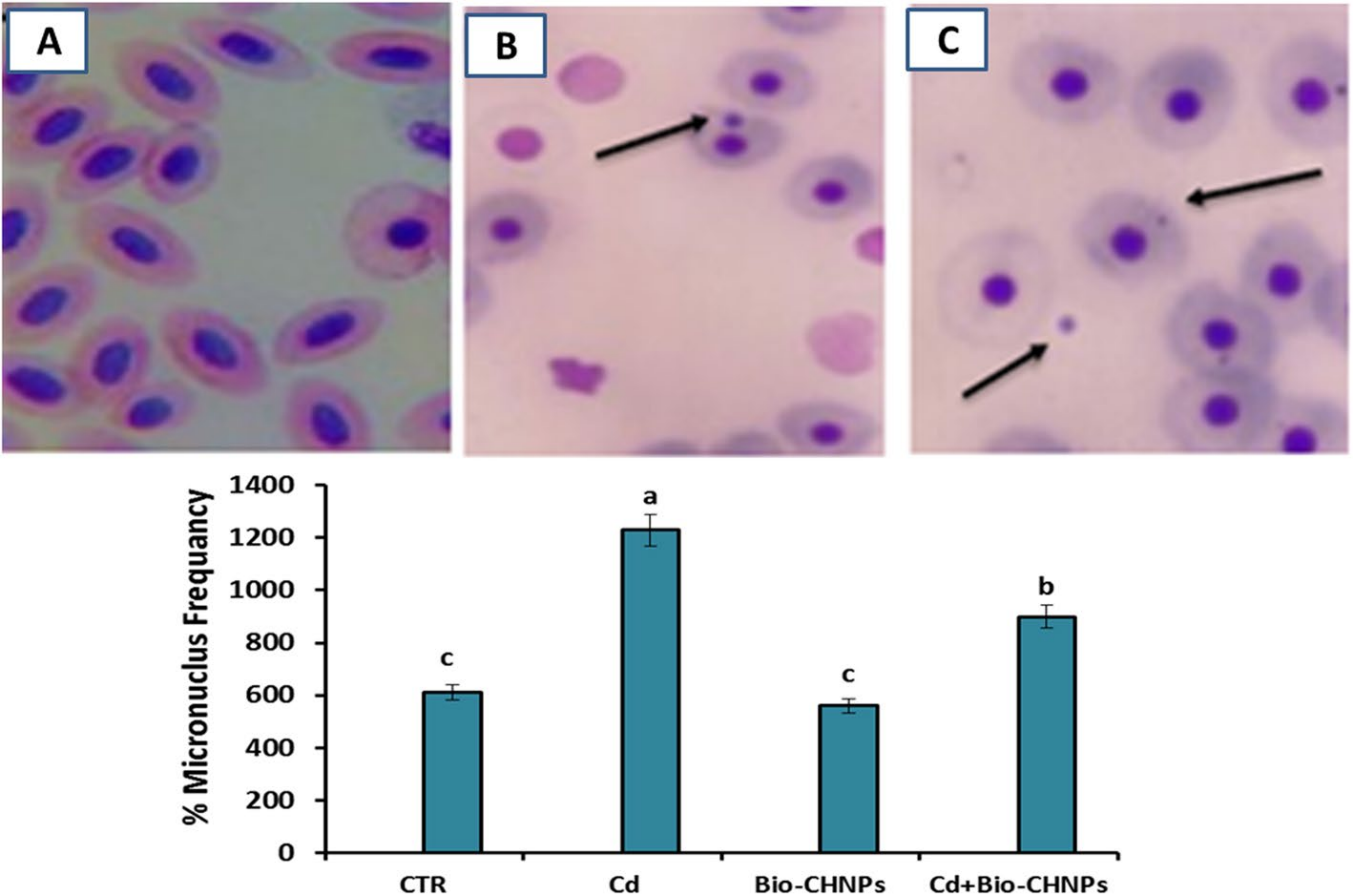
Micronuclei in peripheral blood erythrocytes of catfish exposed to cadmium concentrations. Pictures are illustrative; time of exposure is 8 weeks not detected in CTR (**A**) and increased in (**B**) Cadmium intoxicated group and reduced in Cd treated group with Biological nano chitosan (**C**) nanoparticles by *Bacillus subtilius*

DNA damage in blood, recorded as % of damaged DNA in the tail and whole blood, was elevated in groups exposed to Cd-contaminated water (G2). However, no change or abnormalities were detected between the NC and Bio-CHNPs (G3) groups. Conversely, the percentage of DNA damage in whole blood was reduced in G4 (Fig. 8). Additionally, the frequency of micronuclei formation was elevated in groups exposed to Cd and significantly reduced in groups treated with Bio-CHNPs. No differences were detected among the treated group with Cd and Bio-CHNPs (Fig. 9).

### Histopathological features

**Fig. 10:**
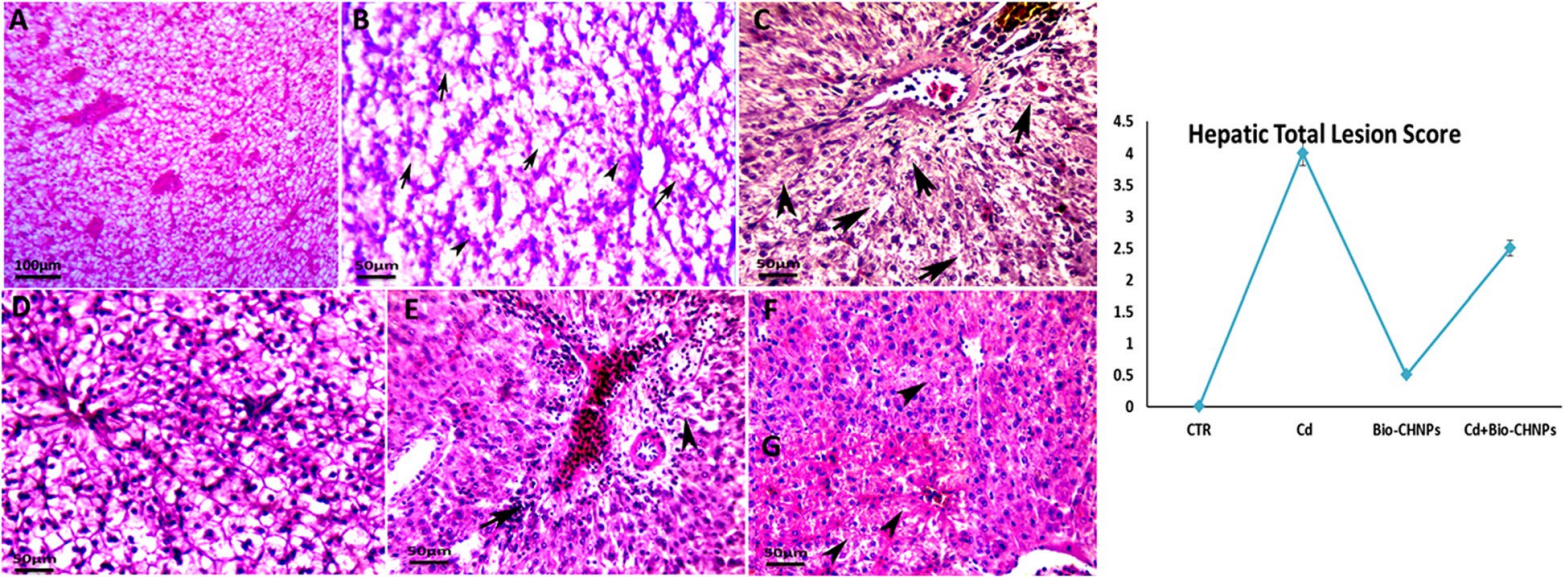
Photomicrographs of the liver sections of catfish. Hepatic tissues of (**A**) normal control (G1), representing normal hepatocytes. (**B**) Cd-treated group (G2) showing degeneration and necrosis of hepatocytes (arrows) with inflammatory cell infiltrations (arrowheads), (**C**) Cd -treated, hepatocyte necrosis (arrows) with degenerated hepatocytes of pyknotic nuclei (arrowheads). (**D**) Bio-CHNPs treated group (G3) showing normal structure of liver tissues. (**E**) Cd + Bio-CHNPs treated group (G4) showing marked improvement in liver tissues represented by a mild degeneration of hepatocytes (arrowheads) with mild inflammatory cell infiltrations (arrows). (**F**, **G**) Cd + Bio-CHNPs treated group (G4) showing marked improvement of hepatocyte regeneration with few cells suffering hydropic degeneration (arrowheads). H&E; scale bar = 50 µm

The hepatocytes and hepatopancreatic arrangement of the Liver of the NC and Bio-CHNPs supplemented groups displayed a normal histological structure. However, a marked diffuse hepatic degeneration with pyknotic nuclei, necrosis, and inflammatory cell infiltrations were observed in the liver of fish exposed to the Cd intoxicated group (Fig. 10). Diffuse hepatic and hepatopancreatic necrosis with cytoplasmic eosinophilia were also noted. In contrast, moderate to complete improvement and restoration of the hepatic tissues with mild inflammatory cell infiltrations were seen in fish exposed to CdCL_2_ and supplemented with Bio-CHNPs (G4).

**Fig. 11:**
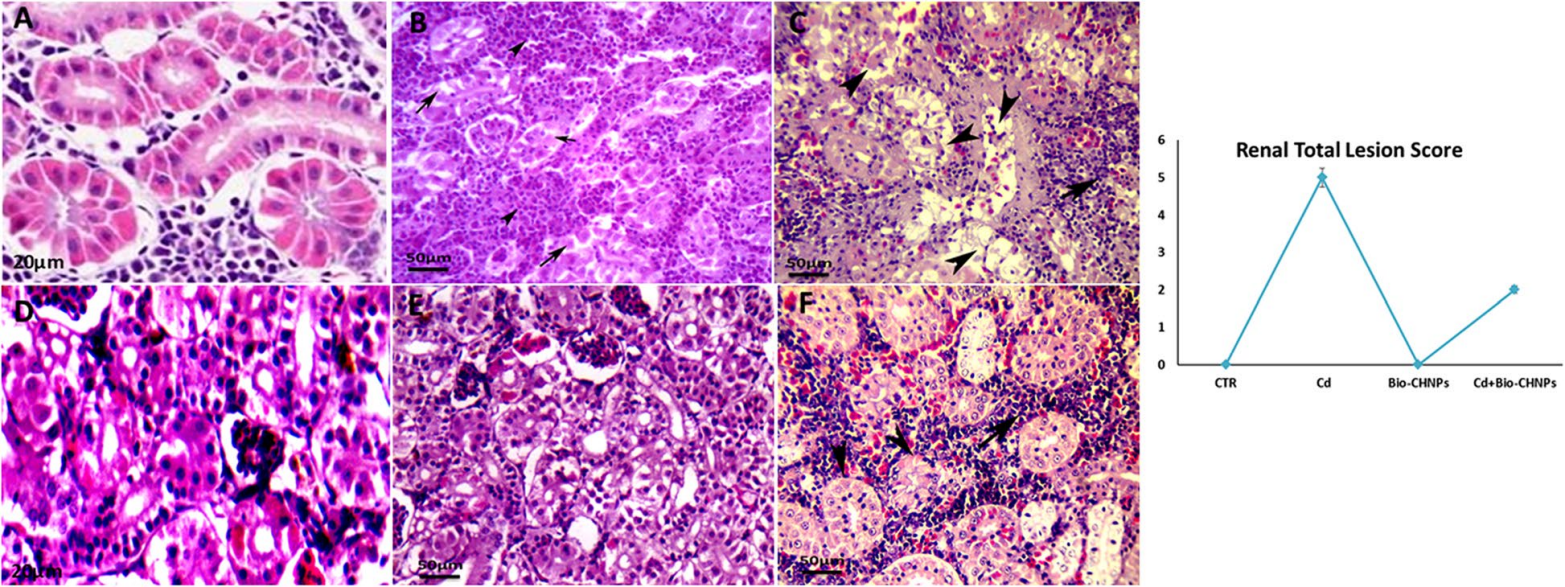
Photomicrographs of kidney sections of catfish. Renal tissues of (**A**) normal control (G1), representing normal kidney tubules. (**B**) Cd-treated group (G2) showing degeneration and necrosis of proximal and distal convoluted tubules (arrows) with severe interstitial leukocytic cell infiltrations between the degenerate and necrosed renal tubules (arrowheads), (**C**) Cd -treated, severe coagulative necrosis of renal tubules (arrowheads) with severe inflammatory cells infiltrations (arrows). (**D)** Bio-CHNPs treated group (G3) showing the normal structure of renal tissues. (**E**, **F**) Cd + Bio-CHNPs treated group (G4) showing marked improvement in renal tissues represented by mild degeneration of renal tubules (arrowheads) and mild inflammatory cell infiltrations (arrows). H&E; scale bar = 50 µm

In kidney sections, a normal renal tubular epithelium of proximal, distal, collecting tubules and glomeruli were seen in the NC and Bio-CHNPs treated group. On the contrary, severe degenerative and necrotic changes of renal tubules with interstitial inflammatory cell infiltrations were observed in renal tissues of fish exposed to Cd toxicity in the G2 group, (Fig. 11). Nevertheless, the fish intoxicated by Cd and treated with Bio-CHNPs expressed moderate to great improvement of renal histological findings indicated by mild degenerative tubular, with mild leukocytic cell infiltrations (Fig. 11).

**Fig. 12:**
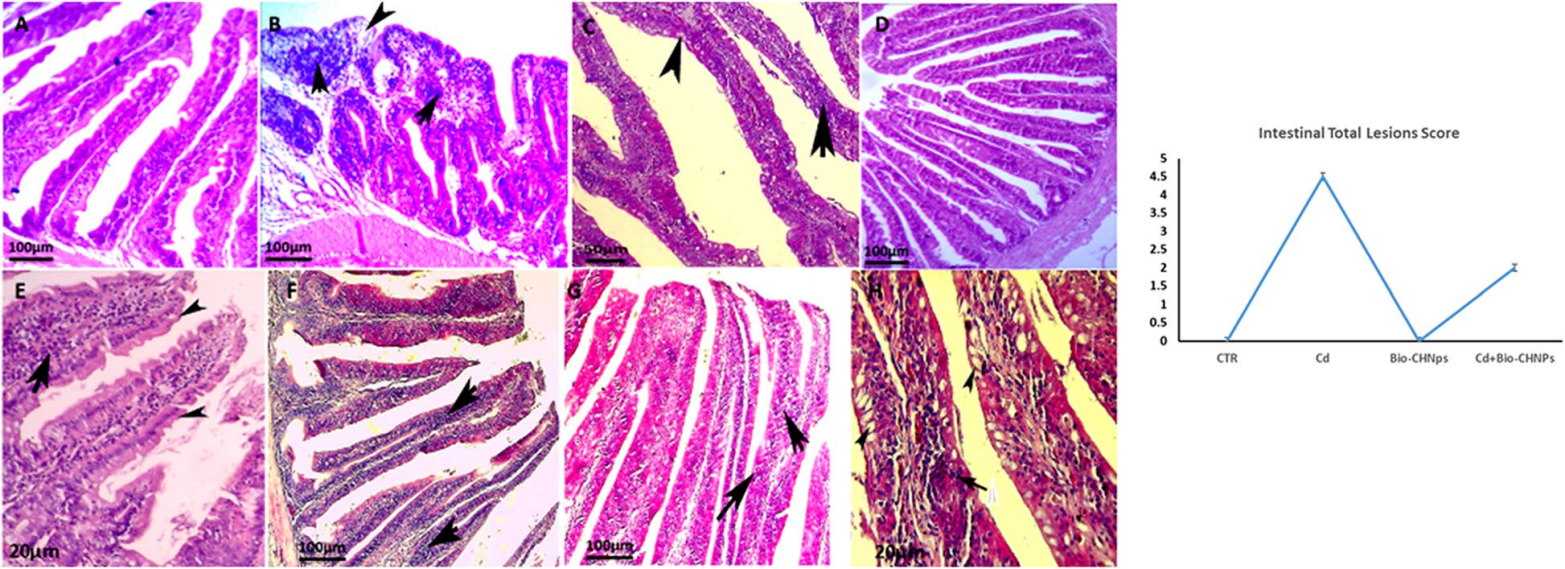
Photomicrographs of intestinal sections of catfish. (**A**) The intestinal tissue of the normal control fish (G1) displayed normal histological structures of the intestinal villi. (**B**) Cd-exposed fish (G2) exhibited severe enteritis, in the form of inflammatory cell infiltrations (arrows) of mucosal and submucosal layers, shortening of intestinal villi with degeneration and necrosis of some columnar epithelial cells (arrowheads). (**C**) Cd-exposed fish (G2) displayed moderate enteritis, with inflammatory cell infiltrations (arrows) and goblet cell hyperplasia (arrowheads). (**D**) Bio-CNPs treated fish (G3) displayed no histopathological changes in the intestinal mucosal layers. (**E**, **F**) Moderate improvement in the intestinal tissues was seen in fish exposed to Cd and treated with Bio-CNPs (G4). (G, H) Moderate to complete restoration and improvement of the intestinal tissues were shown in G4, as mild inflammatory cell infiltration (arrows), and goblet cell hyperplasia (arrowheads) with the increase in the length of intestinal villi

Moreover, the intestinal tissues of the NC (G1) and Bio-CNPs-treated fish (G3) were of normal histological structures of the mucosal, submucosal layer, and intestinal villi, without any pathological alterations. However, CdCL2-exposed fish in G2 displayed moderate to severe enteritis, in the form of inflammatory cell infiltrations of mucosal and submucosal layers, hemorrhage, shortening of intestinal villi, edema with degeneration and necrosis of some columnar epithelial cells (Fig. 12). In contrast, moderate to complete restoration and improvement of the intestinal tissues were shown in G4 as mild inflammatory cell infiltration, and low goblet cell hyperplasia with an increase in the length of intestinal villi. These histopathological findings affirmed the biochemical and immunogenic data, suggesting that the Bio-CNPs supplementation had a substantial protective influence against Cd toxicity in catfish as revealed also by the pathological lesion scoring data of liver and kidney tissues (Figs. 10, 11, 12).

## Discussion

Cadmium is a naturally occurring element, but its levels in water resources increased due to mining, smelting, and industrial processes as human activity. It accumulates and persists in environmental sediments for a long time. A contaminated environment can lead to Cd uptake by plants and fishes which can enter the food chain [47]. As Cd moves up the food chain, it can bioaccumulate in higher-level organism tissue, including humans leading to various health problems [48–50] such as osteoporosis, kidney damage, elevated blood pressure, and cancers of the prostate, lung, breast, and bladder [51–53]. Frequent monitoring of fish tissue contamination by heavy metals is a crucial approach for the early detection of water quality in the related environment and for safeguarding public health [54, 55].

Cd enters the body of fish via various means as gills, skin, and gastrointestinal tract, and enters into the cells due to an extremely high affinity for calcium anion binding sites [55]. In our study, an immense rise in the residue content of Cd in liver and kidney tissues was observed in Cd-intoxicated fish relevant to the other experimented groups. The Cd is absorbed by the fish gills [56], and then stored in the liver and kidney [57]. This finding aligns with the results of Lee et al., [58] who reported the accumulation of Cd in various tissues of carp (*Cyprinus carpio*). The Cd accumulates in fish body organs (muscle, intestine, kidney, liver, and gills) when they are exposed to various forms of Cd [55, 59]. This excellent bioaccumulation is the result of various factors such as the use of fertilizers in aquatic environments that significantly alter water properties reflecting the fish detox system and metabolism [60, 61]. Consequently, the permeation of metal into fish gill and intestinal epithelium is promoted and also the fish could not eliminate Cd, leading to its bioaccumulation in target tissues [62].

The liver is extremely important in detoxifying heavy metals and other xenobiotics because the hepatocytes can synthesize the metallothionein protein that saves the cells by chelating to the Cd ions tightly when the cells are intoxicated with high levels of Cd [55]. A considerable increase in the activities of liver enzymes (AST, and ALT) in the Cd-intoxicated group as compared to the control negative group, indicating liver disease and hepatotoxicity of high levels of Cd [55, 63, 64]. The notable increase in ALT and AST activities can be due to the catalysis of the process of transamination with amino acids for providing energy [65]. AST and ALT, which have vital roles to play in the metabolism of amino acids and proteins [66], are suggested as sensitive ecotoxicological biomarkers for the early detection of adverse changes in stressed aquatic organisms [67].

Blood glucose level, an index of stress response, is secreted as a secondary response to surplus energy after environmental stresses [66, 68, 69]. Glucose fluctuation may be due to liver and kidney injury and protein-energy malnutrition [69, 70]. Herein, fish exposed to high levels of Cd had significantly higher glucose levels compared to NC. This indicates that environmental stressors, such as metal exposures, may affect serum biomarkers such as glucose, total protein, and urea, since their changes may bring severe survival issues to animal health [71].

Oxidative stress is one of the various deleterious effects induced by Cd intoxication [72]. Cd induces oxidative stress with overproduction of free radicals by indirect mechanisms not via generation of ROS directly. Cd can replace copper and iron in the membrane and cytoplasmic proteins of the cells, reflecting on the pump activity of the cellular membrane with a reduction of ATP production [73]. Additionally, redox imbalance enhances oxidative damage of biomolecules such as proteins, lipids, and DNA with a decline in the antioxidant concentration leading to direct adverse effects on the growth, longevity, and reproduction of fish [74, 75]. As antioxidant markers such as SOD, CAT, and TAC levels, have essential roles in free radicals scavenging with prevention of lipid peroxidation damage that is produced by the activation of oxidative stressors such as TRX [76]. Following our outcomes in this study, a significant change in antioxidant parameters and oxidative stress markers was observed in Cd intoxicated fish, as a notable decline in antioxidant markers such as SOD, CAT, and TAC levels, coupled with an increase in the oxidative stress marker (TPX) was detected. The same results were confirmed by the decline in the mRNA expression of the antioxidants (GPX, GSR, and GST) of the liver of cd intoxicated fish. These antioxidants, such as GPX, GSR, and GST have a critical function in enzymatic ROS conversion into less harmful metabolites; thus preventing cellular membrane destruction and restoring cellular defense mechanisms against aquatic toxicity-induced stress [77]. This aligns with other studies that attribute the toxic effect of Cd toxicity to the generation of reactive oxygen species (ROS) and the triggering of oxidative stress by compromising the antioxidant defense mechanisms [78, 79]. The enhanced oxidative stress with ROS generation leads to lipid peroxidation with subsequent DNA damage and strand breaks as noticed in Cd-exposed fish as indicated by alkaline single-cell gel electrophoresis and comet assay [80]. Fragments of DNA and loosened DNA loops migrated towards the anode, forming the tail of a comet, indicating an increase in alkali labile sites and single-strand breaks. Similarly, the Cd induces DNA single-strand breaks in HepG2 cells directly, as demonstrated by Comet assays [80]. The severity of DNA damage is affected directly with the Cd concentration, as it is a dose–response relationship, that increases with the increased Cd dose [80]. Additionally, Cd inhibits DNA repair genes, impairing their repair mechanisms and exacerbating genomic instability as shown in bronchial epithelial cells of human [81].

The respiratory burst is a pillar of innate immunity that enables phagocytic cells like neutrophils and macrophages to kill pathogens by the use of ROS [82, 83]. ROS are vital for eliminating pathogens but become toxic when unregulated [84]. As this process balances pathogens destruction with controlled oxidative stress to protect tissue homeostasis [83]. Further, LYZ degrades the bonds in peptidoglycan, destabilizing the pathogen's cell wall, triggering phagocytic cells and complement system leading to bacterial lysis [85]. In our study, immunosuppression was detected in Cd-intoxicated fish displayed by a marked reduction in RB, LYZ, TP levels, and Ig. Ig plays critical roles in innate immunity, recognizes conserved microbial structures, binds pathogens with activation of the classical complement cascade, removes apoptotic cells, prevents inflammation, enhancing phagocytosis and pathogen clearance within the fish body [86]. The decline in the activities of RB, LYZ, TP, and Ig can be correlated with the suppressive effect of herbicides and heavy metal toxicity on immune response, particularly leukocyte production and differentiation [87]. The decline in the innate immune response and total protein levels, primarily of those synthesized in the liver, could signify liver dysfunction, hepatocyte apoptosis, decreased absorption, and protein loss, and further reflects on the health status of the fish [88]. The health condition of the fish due to Cd intoxication was affected by moderate to severe behavioral disorders with a decrease in the survival rate as compared to the NC.

Inflammatory cytokine screening has become an important tool for assessing immune responses and potential immunotoxicity in fish so studying their gene expression is promptly affected by infection, toxicity, and/or inflammation [89]. In particular concern, IL-1β, IL8, and LBP are selected as markers for assessing the immune response of the fish toward several environmental stressors and other pathogens due to the crucial roles of these genes in immune function regulation [89]. IL-1β is a pro-inflammatory cytokine frequently documented in fish immunotoxicity research [89, 90]. IL-8 is a potent inflammatory cytokine that plays an essential role in the immune response of fish [91]. It is a chemotactic factor that is associated with attraction and activation of neutrophils and other leukocytes to the sites of inflammation and elimination of various fish pathogens which are essential for the innate immune system [91]. More importantly, LBP is a trace plasma protein, that binds to the lipid of bacterial lipopolysaccharides (LPSs), and has a vital role in defending the host against gram-negative bacteria via modulating immune responses and neutralization of LPS toxicity in fish [92]. In the existing study, significant upregulation of the mRNA expression values of the inflammatory-related genes (IL1β, IL8, LBP) was seen in Cd-exposed fish. The inflammatory pathway together with the enhanced oxidative stress are further essential mechanisms contributing to Cd-induced tissue damage in fish [93]. As ROS generation triggers the cascade of intracellular signaling, inflammatory mediators release culminating in the upregulation of the proinflammatory genes and inflammatory response enhancement [94, 95]. Consequently, the decline in fish resistance and immune responses with upregulation of oxidative stress markers corresponded with the degenerative and necrotic pathological changes and leukocytic cell infiltrations in Cd-exposed fish. These pathological findings in the hepato-renal and intestinal tissues are also congruent with the earlier studies [96, 97].

The endocrine system regulates vital physiological functions through hormone-dependent mechanisms. Hormones like progesterone, testosterone, and GH play essential roles in enhancing fish growth. Progesterone is often administered with testosterone in fish farming to optimize the growth, size, and weight of the fish for increased economic gain [98]. Progesterone plays an important role in growth and gonadal development, as documented in zebrafish where it interacts with LH and FSH pathways [98] in which the FSH has a crucial role in regulating reproductive processes [99]. In addition, Cortisol acts as a primary stress hormone in fish, maintaining homeostasis, and has critical roles in physiological responses against environmental stressors like temperature changes, pollutants, or salinity shifts [100].

Metal exposure can significantly disturb the hormone levels and consequently impact cellular and tissue functions [101], as these toxicants may directly affect endocrine tissues or disrupt homeostasis in non-endocrine organs [102]. The significant reductions in the FSH, testosterone, progesterone, and GH in Cd-exposed groups, along with an increase in cortisol, align with findings from [101]. Low FSH concentrations during acute exposures to metals reveal a disruption of the reproduction and early gonad development mechanism in fish species and the hypophyseal–gonadal axis activity dysfunction [102], along with the impairment of the hypophyseal–gonadal axis activity. The hypophyseal–gonadal axis activity dysfunction results in decreased sperm volume, and oocyte degeneration [99]. Cortisol is a reliable biomarker for water pollution in fish as its secretion depends on factors such as pollutant type, time, and duration of exposure. Organic pollutants such as pesticides typically raise cortisol secretion, however, persistent organic pollutants and heavy metals particularly in long-term exposure often suppress cortisol secretion [103].

Chitosan is a natural polysaccharide having many biological properties, capable of adsorbing various aquatic pollutants [104, 105]. Widely, CHNPs have been used in the industry, especially in food, and drug delivery systems, and as carriers for different bioactive agents. CHNPs have been utilized as feed additives to enhance immunity and growth, and also for enhancement of fish resistance against various environmental stressors [106, 107]. CHNPs have inherent anti-inflammatory, antibacterial, antioxidant, anticancer, and hemostatic activities [108–110].

Microorganisms, particularly the *Bacillus* species have been selected as an efficient agent in the biosynthesis of NPs and detoxification of heavy metals [111, 112]. These bacteria offer an efficient and safe potential for the biosynthesis of eco-friendly NPs and reduce the over-costs needed for hazardous chemicals [113]. As outlined in our study, *Bacillus subtilis* pellets were added to the chitosan solution, and the colorless solution was turned to a dark brown color indicating the biosynthesis of Bio-CNPs from the chitosan supplement [114].

The antioxidant and anti-inflammatory activities of CHNPs are the most plausible effective mechanisms contributing to the inclusion of CHNPs against environmental pollutants and the use of pesticides in the eco-environment [115]. Consistently, our research revealed that Bio-CHNPs supplementation could provide a powerful antioxidant effect versus oxidative stress induced by the Cd toxicant as evidenced by elevation of SOD, CAT, and TAC levels upon Bio-CHNPs dietary supplementation, suggesting the protective role of Bio-CHNPs. The protective antioxidant aptitude of Bio-CHNPs against oxidative stress induced by Cd intoxication is confirmed by the ability of Bio-CHNPs to scavenge and inhibit the free radicals using DPPH activity. Bio-CHNPs have potent antioxidant activity and neutralize the existing free radicals and prevent their generation again [116]. In addition, much recent compelling research highlights the inclusion of CHNPs in the food and drug industry referring to their effectiveness as an anti-inflammatory, antibacterial, and antioxidant, besides, their role in the enhancement of immunomodulatory responses [115, 117, 118].

Noteworthy, our research elucidates that Bio-CHNPs supplementation could potentially relieve the clinical signs and pathological alterations displayed by fish intoxicated by Cd, increase the survival rate along with reduction of fish mortality. Besides dramatic enhancement of GH levels and male and female hormones were also observed in the Bio-CHNPs treated group along with a decline of cortisol levels, suggesting a positive impact on hormonal balance. Furthermore, Bio-CHNPs have a role in the transcriptional regulation of immunomodulatory reactions and mitigation of the inflammatory response by obscuring the release of proinflammatory cytokines [119] that are upregulated in Cd-exposed fish.

Notably, the Bio-CHNPs feeding group expressed no detection of cadmium residue [57]. Interestingly, the group supplemented with diets mixed with Bio-CHNPs and Cd-polluted water exhibited a significant decline in Cd residue levels in fish tissue, reaching levels comparable to the fish NC group, This suggests a potential mitigating effect of Bio-CHNPs on Cd accumulation in fish tissue [120].

## Conclusion

Cadmium toxicity poses a serious environmental problem affecting farm soil, water life, and can find its way into the food chain. Our current study highlights the promising avenue of incorporating Bio-CHNPs in alleviating Cd-induced toxicity. The study indicated impressive improvement in using Bio-CHNPs as reflected in its capability of recovering essential physiological functions in affected aquatic organisms. The application of Bio-CHNPs helps in re-establishing hormonal equilibrium, mitigating oxidative stress caused by Cd exposure with the enhancement of the antioxidant defenses, and having a beneficial impact on gene expression, crucial for the health and reproductive quality of aquatic organisms. The results indicate that Bio-CHNPs can be effectively employed to remove Cd from fish tissue, presenting a viable solution for managing environmental pollution. This holds wide-ranging consequences for both environmental protection and public health.

## Supplementary Information

Additional file 1. Supplementary Fig. 1. The inhibition zone diameters of the biological nano-chitosan (Bio-CNPs) with minimum inhibitory concentration (MIC) and minimum bactericidal concentration (MBC) levels against certain Gram-positive (G+) and Gram-negative (G-) pathogenic bacteria with different concentrations 1, 2 and 3).

Additional file 2. Supplementary Table 1. Primer sequences, amplicons, and the related information for quantitative PCR in liver tissue.

## Data Availability

All data sets obtained and analyzed during the current study are available in the manuscript. Further inquiries can be directed to the corresponding author.
