# Supplementary figures and images for: Mitigation of cadmium toxicity in African catfish using biological Nano chitosan: insights into biochemical, genotoxic, and histopathological effects
Source: BMC Vet Res. 2025 Apr 16;21:278. doi: 10.1186/s12917-025-04673-4 (PMC12004861; doi:10.1186/s12917-025-04673-4)

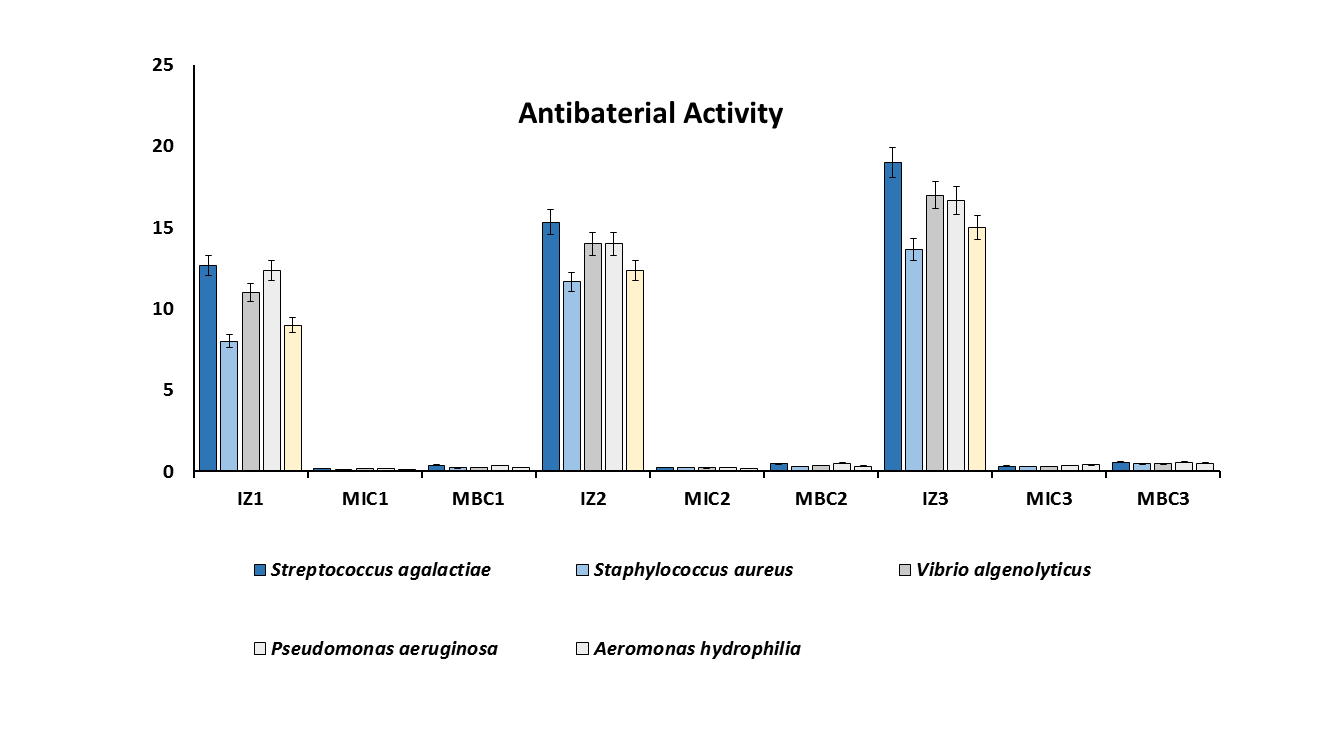

Supplement: Supplementary file 1 — Additional file 1. Supplementary Fig. 1. The inhibition zone diameters of the biological nano-chitosan (Bio-CNPs) with minimum inhibitory concentration (MIC) and minimum bactericidal concentration (MBC) levels against certain Gram-positive (G+) and Gram-negative (G-) pathogenic bacteria with different concentrations 1, 2 and 3). [file 12917_2025_4673_MOESM1_ESM.tif]
