# Supplementary material for: Mitigation of cadmium toxicity in African catfish using biological Nano chitosan: insights into biochemical, genotoxic, and histopathological effects
Source: BMC Vet Res. 2025 Apr 16;21:278. doi: 10.1186/s12917-025-04673-4 (PMC12004861; doi:10.1186/s12917-025-04673-4)
Supplement: Supplementary file 2 — Additional file 2. Supplementary Table 1. Primer sequences, amplicons, and the related information for quantitative PCR in liver tissue. [file 12917_2025_4673_MOESM2_ESM.docx]

**Supplementary Table .1.** Primer sequences, amplicons, and the related information for quantitative PCR in liver tissue.

| **Primer Name** | **Primer Sequence (50ʹ-30ʹ)** | **Target Gene** | **Tm (◦C)** | **Product Size (bp)** | **Accession No.** |
| --- | --- | --- | --- | --- | --- |
| 18S rRN F | GTGCATGGCCGTTCTTAGTT | 18S rRNA | 60 | 150 | XR_003216134 |
| 18S rRNA R | CTCAATCTCGTGTGGCTGAA |  | 60 |  |  |
| IL1 F | GTCTGTCAAGGATAAGCGCTG | IL1 | 59 | 200 | XM_019365844 |
| IL1R | ACTCTGGAGCTGGATGTTGA |  | 58 |  |  |
| IL8 F | CTGTGAAGGCATGGGTGTG | IL8 | 59 | 196 | NM_001279704 |
| IL8 R | ATCACTTTCTTCACCCAGGG |  | 58 |  |  |
| LBP F | ACCAGAAACTGCGAGAAGGA | LBP | 59 | 200 | XM_013271147 |
| LBP R | GATTGGTGGTCGGAGGTTTG |  | 59 |  |  |
| GSTa F | ACTGCACACTCATGGGAACA | GSTa | 60 | 190 | NM_001279635 |
| GSTaR | TTAAAAGCCAGCGGATTGAC |  | 60 |  |  |
| GPX F | GGTGGATGTGAATGGAAAGG | GPX | 60 | 190 | NM_001279711 |
| GPX R | CTTGTAAGGTTCCCCGTCAG |  | 59 |  |  |
| GSR F | CTGCACCAAAGAACTGCAAAC | GSR | 60 | 172 | XM_005467348 |
| GSR R | CAGAGAAGGCAGTCCACTC |  | 60 |  |  |
| IL1β: interleukin 1β, IL8: interleukin 8, LBP: lipopolysaccharide binding protein, GSTa: glutathione S-transferase, GPX: glutathione peroxidase, GSR: glutathione-disulfide reductase. | | | | | |
